# Public Support for Nutrition-Related Actions by Food Companies in Australia: A Cross-Sectional Analysis of Findings from the 2020 International Food Policy Study

**DOI:** 10.3390/ijerph20054054

**Published:** 2023-02-24

**Authors:** Ebony Yin, Adrian J. Cameron, Sally Schultz, Christine M. White, Lana Vanderlee, David Hammond, Gary Sacks

**Affiliations:** 1 Institute for Health Transformation, Global Centre for Preventive Health and Nutrition, Deakin University, Burwood 3125, Australia; 2 School of Public Health Sciences, University of Waterloo, Waterloo, ON N2L 3G1, Canada; 3 School of Nutrition, Centre Nutrition, Santé et Société (NUTRISS), Institut sur la Nutrition et les Aliments Fonctionnels (INAF), Université Laval, Québec City, QC G1V 0A6, Canada

**Keywords:** food industry, food environment, nutrition policy, food company, nutrition initiative, public attitudes

## Abstract

Unhealthy food environments contribute to unhealthy population diets. In Australia, the government currently relies on voluntary food company actions (e.g., related to front-of-pack labelling, restricting promotion of unhealthy foods, and product formulation) as part of their efforts to improve population diets, despite evidence that such voluntary approaches are less effective than mandatory policies. This study aimed to understand public perceptions of potential food industry nutrition-related actions in Australia. An online survey was completed by 4289 Australians in 2020 as part of the International Food Policy Study. The level of public support was assessed for six different nutrition-related actions related to food labelling, food promotion, and product formulation. High levels of support were observed for all six company actions, with the highest support observed for displaying the Health Star Rating on all products (80.4%) and restricting children’s exposure to online promotion of unhealthy food (76.8%). Findings suggest the Australian public is strongly supportive of food companies taking action to improve nutrition and the healthiness of food environments. However, given the limitations of the voluntary action from food companies, mandatory policy action by the Australian government is likely to be needed to ensure company practices align with public expectations.

## 1. Introduction

Unhealthy diets are a key risk factor for non-communicable diseases (NCDs) and a global health priority [1]. It is widely accepted that food environments have a major influence on dietary intake [2,3]. In Australia, food environments generally do not promote healthy eating [4,5,6,7], with “discretionary” foods that are high in energy, sugar, salt and/or saturated fat widely available and heavily promoted [7]. The supply and marketing of discretionary food in Australia is led by a relatively small number of large food companies with substantial market power [7,8]. These food companies use a wide range of strategies to influence consumers as part of integrated marketing campaigns, including: traditional and digital marketing tactics (e.g., television and outdoor advertisements, social media and gamification) [9]; retail-based promotion (e.g., price promotions, positioning and shelf space) [8]; and on-package marketing (e.g., cartoon characters and health claims) [10,11].

There have been consistent calls for government-led policy action to improve the healthiness of food environments as part of efforts to address unhealthy diets [2,3,12]. Some countries have implemented a suite of mandatory food-related policies including: restricting exposure of children to marketing of unhealthy food [13]; providing front-of-pack nutrition labelling [14]; and increasing the prices of unhealthy foods (e.g., taxes on sugary drinks) [15]. In contrast, the Australian government’s policy response to unhealthy diets falls far short of global benchmarks [16]. Currently, Australia’s nutrition-related policies rely heavily on voluntary action by food companies, including the voluntary Health Star Rating (HSR) front-of-pack nutrition labelling system [17], industry codes for adult and children’s marketing guidelines [18], and the Healthy Food Partnership Reformulation Program [19]. The lack of mandatory action has been attributed to multiple factors, including food industry lobbying to limit regulations that may harm their profits, and the prioritisation of economic wealth over public health [20,21,22,23]. Reliance on voluntary action has for the most part been shown to be ineffective, with limited uptake of such policies by food companies coupled with weak or incomplete implementation where there is uptake [24,25,26]. A 2018 assessment of Australian food company nutrition-related policies and commitments found that most companies fell short of global recommendations [27].

In the absence of government regulation, pressure on food companies from external stakeholders such as the general public and investors can lead to increased implementation of nutrition-related actions (e.g., via corporate sustainability strategies) [28,29,30]. An understanding of the extent of public support for food company action is an important advocacy tool to inform strategies to influence food industry efforts to improve the healthiness of Australian food environments. Public expectations of food companies can also guide government policy development [31].

Previous research has found that public support for various nutrition-related policies differs between countries, due to factors such as differing cultural norms, political ideology, and stage of implementation [32,33]. Research examining public support for nutrition-related policies in Australia has largely focused on support for government-led policy solutions [34,35], with limited research focused on public perceptions related to food company action [36,37,38]. Two previous studies investigated public perceptions of unhealthy food sponsorship at community events and in community sport [37,38]; and one study investigated the perceived responsibility of food companies to address population health outcomes, generally [36]. While these studies found strong support for increased food company action to improve population diets, they were very limited in the scope of the nutrition-related actions they explored. To contribute to addressing this knowledge gap, this study aimed to understand public support for food company actions targeting front-of-pack nutrition labelling, exposure of children to marketing of unhealthy foods and product reformulation in Australia, and how the level of support varied by socio-demographic factors.

## 2. Materials and Methods

### 2.1. Study Design and Sampling

Data are from the 2020 International Food Policy Study (IFPS), an online annual repeat cross-sectional survey conducted across five countries: Australia, Mexico, Canada, the USA, and the UK [39]. The current study used data collected between November and December 2020 from respondents in Australia.

Participants aged 18 to 100 residing in Australia were recruited through Nielsen Consumer Insights Global Panel and their partners’ panels, using non-probability sampling methods. Email invitations were sent to a random sample of eligible panellists. Participants provided informed consent prior to survey completion. Participants received remuneration in line with the panels’ existing incentive structure (e.g., points-based or monetary) [40]. The study received ethics clearance through a University of Waterloo Research Ethics Committee (ORE# 30829). Deakin University Human Research Ethics Committee provided an ethics exemption in 2018. A full description of the study methodology has been published elsewhere [40].

### 2.2. Measures

#### 2.2.1. Support for Food Company Action

Public support was assessed for six actions food companies can take to improve the overall healthiness of the food supply, as outlined in Table 1. The set of actions was derived from global, nutrition-related recommendations for food companies [27]. Respondents were randomly selected to answer only one of the six questions to reduce overall survey length and response fatigue. Support was measured by asking respondents, “Please tell us whether you agree or disagree with the following statement”. A 5-point Likert scale was used to assess support including “strongly agree”, “agree”, “neutral”, “disagree” and “strongly disagree”. Each question also had a “refuse to answer” and “don’t know” option.

#### 2.2.2. Sociodemographic Variables

Self-reported demographic variables included age group (18–29, 30–44, 45–59, 60+ years), sex, education, body mass index (BMI), household income, whether respondents had children, and the respondents’ food shopping responsibility. Education was categorised into three levels; “low” (year 12 or lower), “medium” (trade certificate or diploma) and “high” (bachelor’s degree or above). BMI was calculated using self-reported height and weight and was categorised according to World Health Organization classification [41]. Household income was reported in ranges of AUD 10,000 from “Less than AUD 10,000” to “AUD 150,000 and over”. Equivalised household income was calculated using the OECD-modified equivalence scale [42]. This scale is used by the Australian Bureau of Statistics to adjust for economies that occur from sharing resources within households, allowing for more meaningful comparisons of household income [43]. The equivalisation scale assigns a value of 1 to the household head, 0.5 to each additional adult and 0.3 to each child [42]. The categorical data collected for income were assigned a value in the middle of each income range (e.g., AUD 20,000–30,000 became AUD 25,000). The OECD-modified equivalence scale was applied to this value to determine an estimated equivalised household income. Income was then recategorized into low, medium, and high tertiles. Variables representing socio-demographic characteristics were selected for inclusion in regression models a priori based on being both assessed in the IFPS study and known to influence diet-related behaviours [32,44,45].

The extent of food shopping responsibility was categorised as “most”, “shared equally”, “some, but less than others” and “none”. Dietary health was categorised as “poor”, “fair”, “good”, “very good” and “excellent”. Each variable also had “refuse to answer” and “do not know” options.

### 2.3. Data Management and Analysis

A total of 5500 respondents completed the survey. Respondents were excluded for the following reasons: invalid response to a data quality question; survey completion time under 15 min; and/or invalid responses to at least 3 of 21 open-ended measures (*n* = 1211), leaving an analytic sample of 4289 respondents. Participants with missing results for the sociodemographic variables were included in the descriptive analysis, but were excluded in the logistic regression models that included these variables. Missing data, “refuse to answer”, and “do not know” responses were excluded from analysis. Data were weighted using post-stratification sample weights constructed using a raking algorithm with population estimates based on age, sex at birth, region, ethnicity, and education [40]. Estimates reported are weighted. Analyses were conducted using Stata/BE-17 [46].

Explanatory variables used in the models included age, sex, BMI, education, equivalised household income, shopping role, guardian/parental status, and health of diet. These were chosen as covariates based on the existing literature [34,44].

Additional sensitivity analysis was undertaken to determine best fit of the model through exploratory univariate logistic regression modelling for each covariate [47]. To determine the impact of “neutral” responses, a separate multivariable logistic regression analysis was conducted on all outcome measures, excluding “neutral” responses. The results from this analysis were similar to the final model that included the “neutral” response option. The final model was tested for goodness of fit using the Hosmer–Lemeshow test [47]. Due to the number of response options being tested, the significance level was set at the 0.01 level.

## 3. Results

### 3.1. Sample Characteristics

The weighted sociodemographic characteristics of respondents are detailed in Table 2. The mean age of respondents was 46.6 years (min 18–max 92) and there was an approximately equal proportion of male and female respondents. The majority of respondents reported low to medium education levels, having no children, doing most of the food shopping in their household and rated their overall diet quality as “good” to “excellent”.

### 3.2. Support for Food Company Action

**Figure 1:**
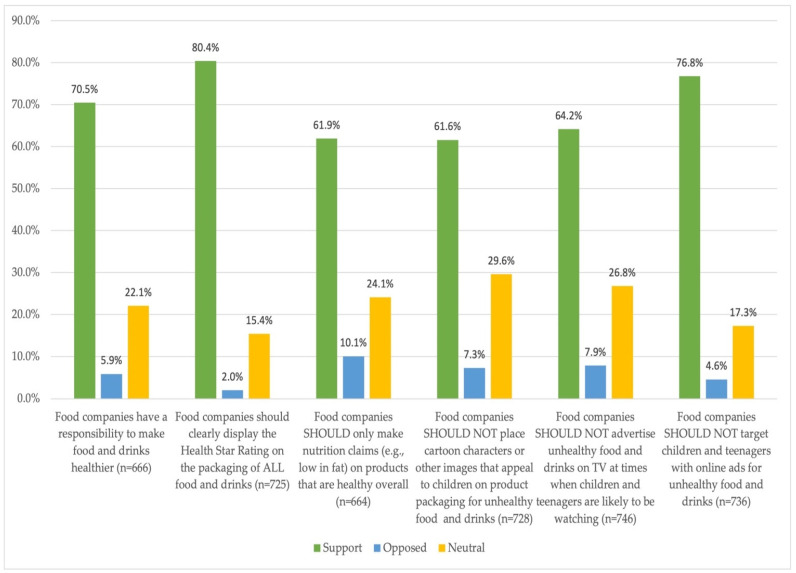
Proportion of Australian public support for nutrition-related actions by food companies (%), IFPS, 2020. Weighted data used for total number of respondents in each category.

The proportion of respondents who supported the various food company nutrition-related actions is detailed in Figure 1. There was more than 60% support for all actions, with the highest level of support for food companies displaying the Health Star Rating on packaging of all food and drinks (80.4%). The lowest support was for food companies not placing “cartoon characters or other images that appeal to children on product packaging for unhealthy food and drinks” (61.6%) and only making “nutrition claims on products that are healthy overall” (61.9%). Across all food company actions, the proportion of participants who opposed the actions was low (2.0% to 10.1%), while the proportion of participants reporting a neutral response ranged from 15.4% to 29.6%.

### 3.3. Support for Food Company Actions by Sociodemographic Characteristics

Results from the multivariable logistic regression model fitted to examine associations between sociodemographic characteristics and level of support for voluntary food company action are detailed in Table 3. Overall, age was a significant covariate for three of the six initiatives. Respondents aged over 60 years old were more than twice as likely than 18–29 year-olds to support food companies “not placing cartoon characters or other images that appeal to children on product packaging for unhealthy food and drinks”, and “not advertising unhealthy food and drinks on TV at times when children and teenagers are likely to be watching”. Those aged above 60 years were more than three times as likely than 18–29 year olds to support food companies “not targeting children and teenagers with online ads for unhealthy food and drinks”. No significant differences in support were found for any other age groups.

Females were almost twice as likely as males to report support for not targeting “children and teenagers with online ads for unhealthy food and drinks”. Sex was not significantly associated with support for any other initiative. Respondents with bachelor’s degrees or above were more than twice as likely to support food companies not targeting “children and teenagers with online ads for unhealthy food and drinks” compared to respondents with low education levels.

No significant associations were found between categories of household income, BMI, parental status, shopping responsibility, and the overall health of diet and level of support for any initiative. For three food company initiatives (that food companies “have a responsibility to make food and drinks healthier for consumers”, “should clearly display the Health Star Rating on the packaging of ALL food and drinks” and “should only make nutrition claims on products that are healthy overall”), no significant associations were found between any sociodemographic variables or BMI and level of support.

## 4. Discussion

This study found strong public support for food companies to take action to improve the healthiness of Australian food environments. The highest level of support was observed for displaying the Health Star Rating on all products, restricting exposure of children to promotion of unhealthy food online, and manufacturing healthier food and drinks. Support for restricting other types of marketing of unhealthy products to children and the responsible use of nutrition claims was also high.

Public support for voluntary nutrition-related action by food companies in this study was generally consistent with findings related to the support of government regulation of food companies from previous studies in Australia and internationally [33,34,35,37,38,48]. A scoping review of 18 studies that explored Australians’ views on regulatory nutrition policies found high levels of support for implementation of interpretive front-of pack nutrition labelling, and moderate to high levels of support for restricting unhealthy food marketing to children and reformulation to improve product healthiness [35]. Likewise, an international study examining public support for nutrition interventions in seven countries, including Australia, found high support across all countries for reformulation interventions and interpretive front-of-pack nutrition labelling (e.g., Health Star Rating, Nutriscore) [48].

The strong level of support for Health Star Rating labelling corresponds with previous studies that have found support for health-related policies and actions increased after their widespread implementation [32,49]. In Australia, the Health Star Rating system was first introduced in 2014, with uptake increasing to 43% of eligible products by 2021 [7]. Some studies have posited that increased acceptance of an initiative after implementation may be associated with the public observing positive impacts or not observing negative consequences [49].

The association between demographic characteristics and the extent of support for various food company nutrition-related actions was generally uniform, with some variation across the different actions. Of note, support for food companies not targeting children with online advertisements for unhealthy food and drinks was significantly higher for those over 60 years compared with 18–29 year olds. Other studies have also found that those above 60 years old were more likely to support nutrition-related policies that were similar to the ones examined in this study [33,50]. The lack of association between parental status and support for food company actions is consistent with previous research which found that parental status was not significantly associated with support for government policies focused on restricting the marketing and promotion of unhealthy food and beverages to children [37,50,51]. While previous literature has identified being female and having a higher level of education as common demographic characteristics associated with increased support for food-related interventions (i.e., sugar sweetened beverage tax, food placement, price-promotion, and restriction of unhealthy food marketing to children), the current study found no significant association between education and most nutrition-related actions [34,44,50]. The exception was a significant association between education and support for online advertising restrictions. The lack of significant differences in the results across different socioeconomic groups likely reflects the broad support for such measures across the population.

Despite this study’s findings that there is both strong public support for companies to take action to improve nutrition, and minimal public opposition to such action, voluntary uptake of globally recommended nutrition-related actions by food companies in Australia has generally been limited. The most recent report (2020) measuring uptake of the Health Star Rating system showed that, six years post-implementation, only 41% of eligible products displayed the Health Star Rating [7]. Reformulation efforts have also been limited, with little change in the overall nutritional quality across all packaged food categories between 2019 and 2021, and few companies formally committing to the Healthy Food Partnership’s reformulation program [7]. There is also consistent evidence to demonstrate the inadequacy of current industry self-regulation in protecting Australian children from unhealthy food marketing online, on television, outdoors, and through sport sponsorships [52,53,54,55]. An assessment of Australia’s largest food and beverage manufacturers found there were significant opportunities to improve nutrition-related policies and practices across the sector, including those related to reformulation, nutrition labelling, and food marketing [27].

### Implications

Overall, the relatively low level of implementation of globally recommended nutrition policies by food companies likely indicates that public support for nutrition-related action is not sufficient to drive policy and practice change for the food industry as a whole. Nevertheless, there appears to be potential to capitalise on the high levels of public support for action to better advocate for change by food companies. Such advocacy is likely to prove most influential if it involves coalitions working together [3]. Due to their potential to influence the actions of public companies, including the large multi-national food companies that dominate food systems in Australia, the institutional investment community may represent a potential lever for increased action [56].

The Australian government currently relies heavily on voluntary actions to improve population diets. Not only do such policies fall short of global recommendations, over the past five years (2017–2022) little policy progress has been observed at the federal government level [16]. The recently released National Obesity Strategy (2022–2032) [57] and National Preventive Health Strategy (2021–2030) [58] have a strong focus on policies for creating healthier food environments, including in the areas of food labelling, food promotion, and food composition. Public support for food company actions in this area is an important consideration as part of policy development processes [21], with the current study indicating strong public support for greater action. A number of other countries, including the United Kingdom [59] and Chile [60], have recently implemented mandatory regulations in these areas, providing a clear pathway for action for the Australian government.

The findings from the current study provide important insight into the current perceptions of the Australian public towards nutrition-related actions by the food industry. The study’s main strength is that it drew data from a relatively large sample of Australians (with selection of participants weighted to ensure the sample closely resembled the population sociodemographics in Australia). Respondents were recruited using nonprobability-based sampling from a commercial panel, meaning that despite the national sample, the findings should not be presumed to provide nationally representative estimates [61,62]. Importantly, the survey measures did not specify whether the relevant food company action would be implemented voluntarily or in response to government legislation. As such, this study is not able to provide any indication of whether the Australian public prefers a voluntary or mandatory approach to food company nutrition-related actions [63].

## 5. Conclusions

This study found strong public support in Australia for food companies to take action to improve nutrition and the healthiness of food environments. The findings from this study support greater implementation of nutrition-related policies and initiatives focused on improving the healthiness of food products, transparent labelling practices and socially responsible marketing strategies. With the current reliance on voluntary action from food companies in Australia, mandatory policy action may be needed to ensure company practices align with public expectations.

## Figures and Tables

**Table 1:** Voluntary food company actions assessed in the IFPS study, 2020.

Food composition	Food companies have a responsibility to make food and drinks healthier for consumers (e.g., by reducing salt/sugar/saturated fat).
Food labelling	Food companies SHOULD clearly display the Health Star Rating on the packaging of ALL food and drinks. Food companies SHOULD only make nutrition claims (e.g., low in fat) on products that are healthy overall.
Food Promotion	Food companies SHOULD NOT place cartoon characters or other images that appeal to children on product packaging for unhealthy food and drinks. Food companies SHOULD NOT advertise unhealthy food and drinks on TV at times when children and teenagers are likely to be watching. Food companies SHOULD NOT target children and teenagers with online ads for unhealthy food and drinks.

**Table 2:** Sociodemographic characteristics of Australian IFPS respondents, 2020 (*n* = 4289).

**Sex**	
Male	49.1%
Female	51.0%
**Age**	
18–29	21.1%
30–44	26.9%
45–59	23.9%
60+	28.1%
**Education**	
Low	41.9%
Medium	32.0%
High	25.5%
Not stated	0.5%
**Household Income**	
Less than AUD 10,000	3.0%
AUD 10,000 to less than AUD 20,000	4.7%
AUD 20,000 to less than AUD 30,000	11.6%
AUD 30,000 to less than AUD 40,000	8.8%
AUD 40,000 to less than AUD 50,000	8.0%
AUD 50,000 to less than AUD 60,000	9.1%
AUD 60,000 to less than AUD 70,000	7.3%
AUD 70,000 to less than AUD 80,000	5.7%
AUD 80,000 to less than AUD 90,000	5.3%
AUD 90,000 to less than AUD 100,000	5.3%
AUD 100,000 to less than AUD 150,000	13.9%
AUD 150,000 and over	8.7%
Not stated	8.6%
**BMI (kg/m^2^)**	
<18.5–24.9	35.8%
25–29.9	26.9%
>30	21.9%
Missing data	15.5%
**Parental Status **	
No Children	58.4%
Has Children	41.6%
Not stated	0.1%
**Amount of food shopping responsibility**	
None	2.5%
Some	7.3%
Equal	24.7%
Most	65.3%
Not stated	0.3%
**Health of Diet**	
Poor	5.8%
Fair	23.9%
Good	44.3%
Very Good	20.9%
Excellent	3.8%
Not stated	1.4%

**Table 3:** Results * from the multivariable logistic regression model (OR, 99% Confidence Intervals **) for support of a range of nutrition-related actions by food companies, IFPS, 2020.

	Food Companies Have a Responsibility to Make Food and Drinks Healthier for Consumers (e.g., by Reducing Salt/Sugar/ Saturated Fat).	Food Companies Should Clearly Display the Health Star Rating on the Packaging of ALL Food And Drinks.	Food Companies Should Only Make Nutrition Claims (e.g., Low in Fat) on Products That Are Healthy Overall.	Food Companies Should Not Place Cartoon Characters or Other Images That Appeal to Children on Product Packaging for Unhealthy Food and Drinks	Food Companies Should not Advertise Unhealthy Food and Drinks on TV at Times When Children and Teenagers Are Likely to Be Watching.	Food Companies Should Not Target Children and Teenagers with Online ads for Unhealthy Food and Drinks.
	OR, [99% CI]	OR, [95% CI]	OR, [95% CI]	OR, [95% CI]	OR, [95% CI]	OR, [95% CI]
Sex						
Male	Reference	Reference	Reference	Reference	Reference	Reference
Female	1.02 (0.57, 1.83)	1.22 (0.73, 2.04)	1.22 (0.73, 2.04)	1.38 (0.83, 2.29)	0.88 (0.54, 1.46)	**1.85 (1.01, 3.41)**
Age						
18–29	Reference	Reference	Reference	Reference	Reference	Reference
30–44	0.85 (0.37, 1.97)	1.14 (0.50, 2.62)	1.00 (0.47, 2.11)	1.32 (0.65, 2.66)	1.63 (0.76, 3.51)	1.13 (0.48, 2.68)
45–59	1.05 (0.45, 2.44)	1.59 (0.58, 4.33)	1.28 (0.57, 2.88)	1.20 (0.58, 2.49)	1.86 (0.85, 4.08)	1.41 (0.58, 3.44)
60+	2.22 (0.88, 5.57)	1.58 (0.60, 4.14)	1.49 (0.65, 3.41)	**2.75 (1.24, 6.12)**	**2.72 (1.24, 5.95)**	**3.49 (1.38, 8.81)**
Education Level						
Low	Reference	Reference	Reference	Reference	Reference	Reference
Medium	1.51 (0.79, 2.90)	0.86 (0.43, 1.70)	1.41 (0.79, 2.51)	1.08 (0.61, 1.94)	0.89 (0.52, 1.54)	1.41 (0.69, 2.89)
High	0.98 (0.46, 2.12)	1.07 (0.48, 2.39)	1.59 (0.78, 3.26)	0.74 (0.38, 1.41)	1.22 (0.62, 2.42)	**2.36 (1.06, 5.22)**
Equivalised Household Income						
Low	Reference	Reference	Reference	Reference	Reference	Reference
Medium	1.06 (0.55, 2.04)	0.64 (0.31, 1.33)	1.15 (0.63, 2.09)	1.36 (0.73, 2.54)	1.34 (0.76, 2.36)	0.74 (0.34, 1.63)
High	1.5 (0.71, 3.17)	0.86 (0.41, 1.82)	0.96 (0.51, 1.79)	1.35 (0.73, 2.50)	1.61 (0.84, 3.06)	0.59 (0.26, 1.34)
BMI (kg/m^2^)						
≤24.9	Reference	Reference	Reference	Reference	Reference	Reference
25–29.9	0.96 (0.45, 2.02)	1.03 (0.49, 2.14)	0.88 (0.47, 1.67)	1.57 (0.82, 2.99)	1.17 (0.63, 2.17)	1.15 (0.55, 2.40)
>30	0.64 (0.30, 1.36)	1.16 (0.49, 2.76)	1.35 (0.67, 2.72)	1.16 (0.60, 2.25)	1.08 (0.57, 2.06)	1.21 (0.53, 2.77)
Missing data	0.49 (0.21, 1.14)	0.68 (0.27, 1.73)	1.25 (0.59, 2.65)	0.72 (0.32, 1.61)	0.99 (0.45, 2.17)	0.68 (0.28, 1.67)
Parental Status						
No Children	Reference	Reference	Reference	Reference	Reference	Reference
Children	0.79 (0.44, 1.43)	0.57 (0.30, 1.10)	1.22 (0.70, 2.14)	0.97 (0.57, 1.63)	1.25 (0.73, 2.12)	0.88 (0.47, 1.67)
Amount of food shopping responsibility						
Never	Reference	Reference	Reference	Reference	Reference	Reference
Some	1.38 (0.21, 9.04)	2.43 (0.37, 15.92)	1.88 (0.34, 10.45)	0.92 (0.09, 9.63)	0.94 (0.18, 4.93)	0.41 (0.04, 4.34)
Equal	2.15 (0.39, 11.87)	2.70 (0.53, 13.83)	1.95 (0.45, 8.43)	1.25 (0.13, 11.79)	0.63 (0.14, 2.82)	0.68 (0.07, 6.58)
Most	3.93 (0.73, 21.12)	3.08 (0.61, 15.53)	2.34 (0.56, 9.83)	1.64 (0.18, 15.17)	1.36 (0.31, 5.99)	0.91 (0.10, 8.43)
Health of Diet						
Poor	Reference	Reference	Reference	Reference	Reference	Reference
Fair	1.27 (0.32, 5.03)	0.28 (0.04, 2.09)	0.56 (0.19, 1.61)	1.14 (0.40, 3.24)	0.57 (0.16, 2.02)	1.26 (0.39, 4.05)
Good	1.43 (0.38, 5.38)	0.24 (0.03, 1.78)	0.79 (0.29, 2.20)	0.92 (0.33, 2.59)	0.70 (0.20, 2.38)	1.33 (0.44, 4.02)
Very Good	1.91 (0.46, 8.04)	0.24 (0.03, 1.93)	1.34 (0.43, 4.20)	1.01 (0.34, 3.04)	0.92 (0.25, 3.39)	1.80 (0.54, 6.07)
Excellent	1.00 (0.15, 6.47)	0.34 (0.02, 4.66)	2.26 (0.40, 12.84)	2.49 (0.50, 12.46)	1.51 (0.24, 9.33)	3.66 (0.44, 30.39)

* Weighted data used for total number of respondents in each category. ** Statistically significant associations (*p* < 0.01) denoted in bold.

## Data Availability

Data are available upon reasonable request to the International Food Policy Study team (see www.foodpolicystudy.com), accessed on 31 January 2023.
